# Supplementary figures and images for: Desert Hedgehog mediates stem Leydig cell differentiation through Ptch2/Gli1/Sf1 signaling axis
Source: eLife. 2026 Jun 29;15:RP109979. doi: 10.7554/eLife.109979 (PMC13313686; doi:10.7554/eLife.109979)

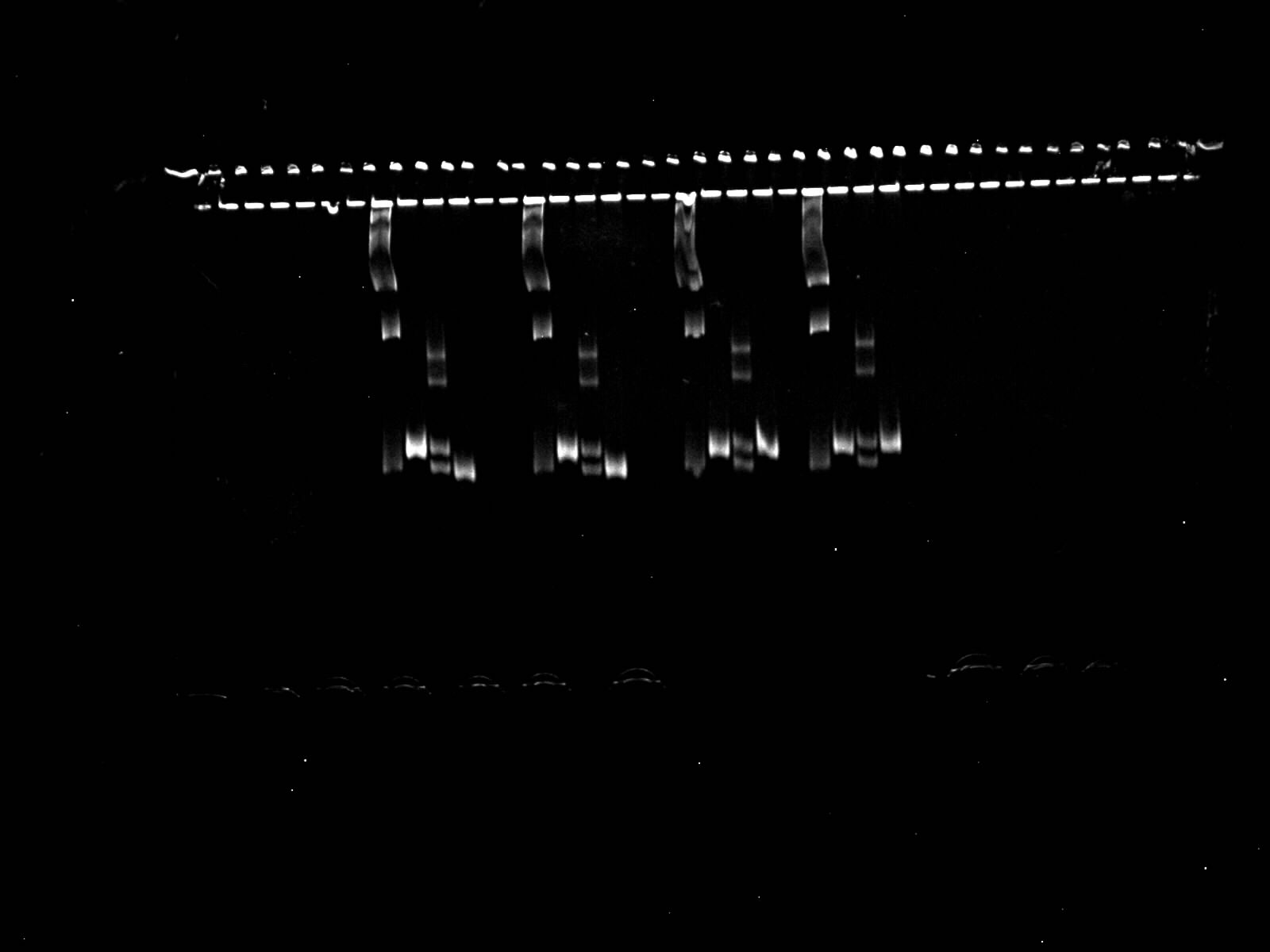

Supplement: Figure 1—figure supplement 2—source data 1. [file elife-109979-fig1-figsupp2-data1.zip › Figure 1-figure supplement 2-source data 1.JPG]

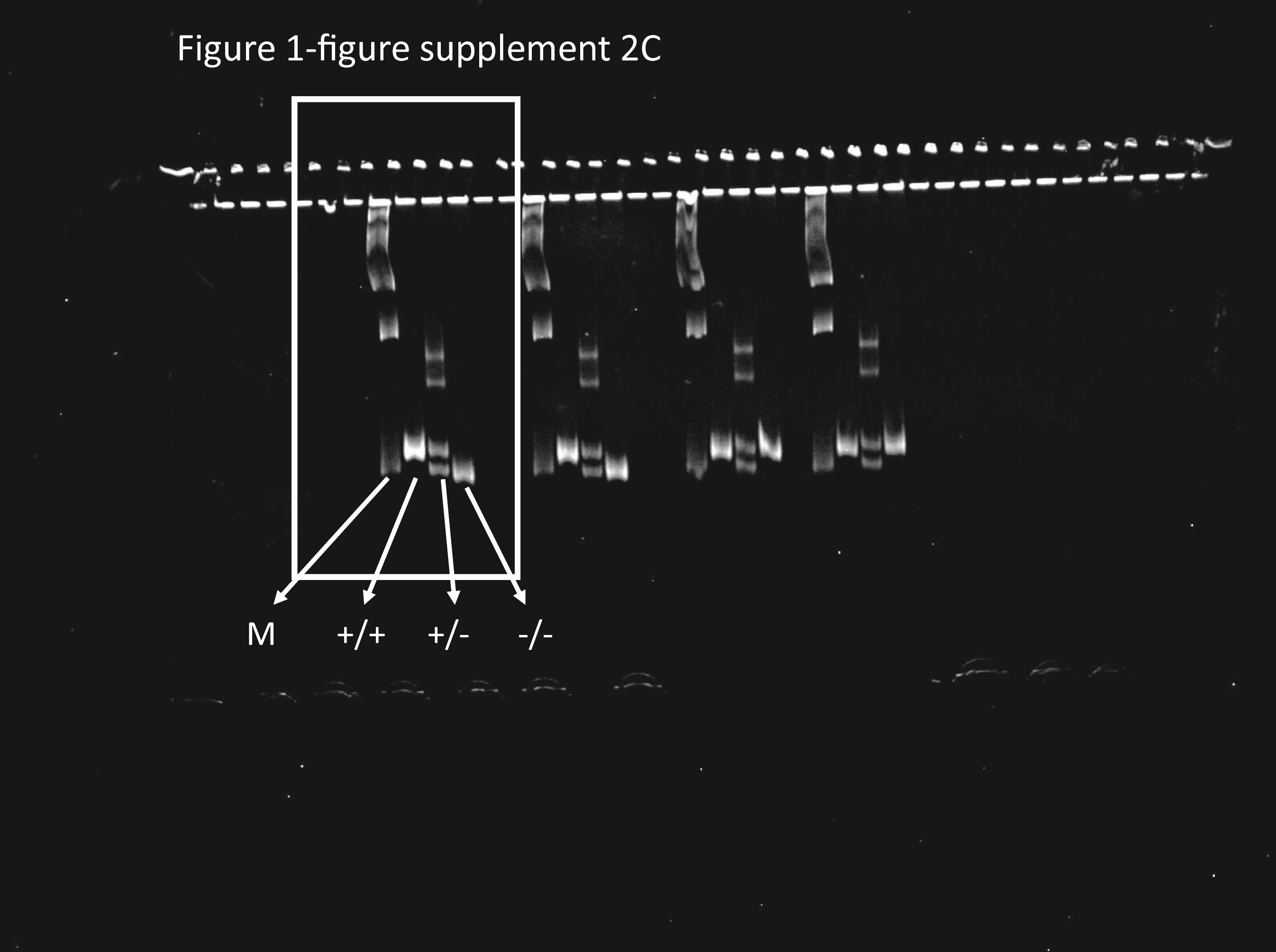

Supplement: Figure 1—figure supplement 2—source data 2. [file elife-109979-fig1-figsupp2-data2.zip › Figure 1-figure supplement 2-source data 2.tif]

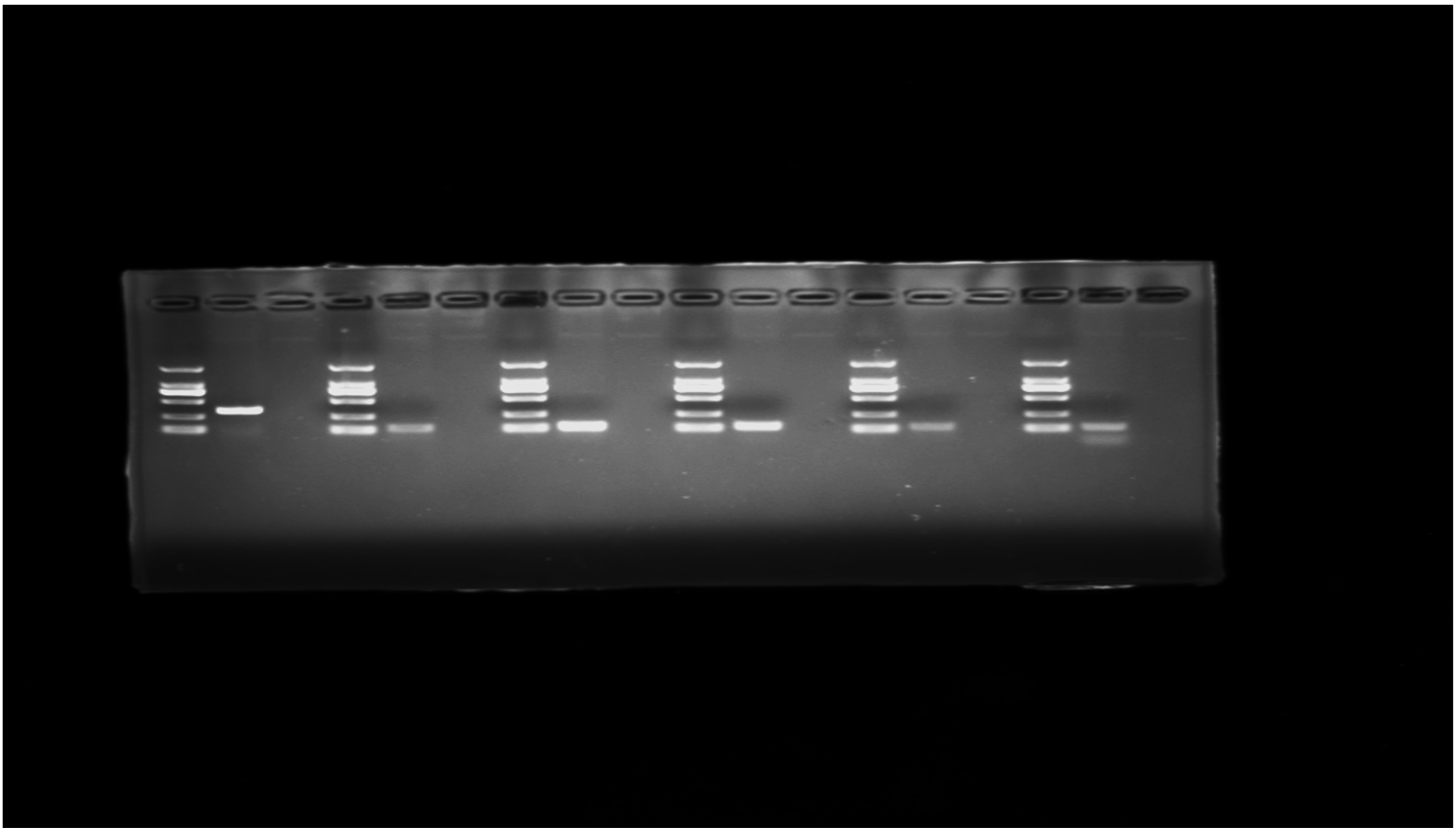

Supplement: Figure 4—figure supplement 1—source data 1. [file elife-109979-fig4-figsupp1-data1.zip › Figure 4-figure supplement 1-source data 1.png]

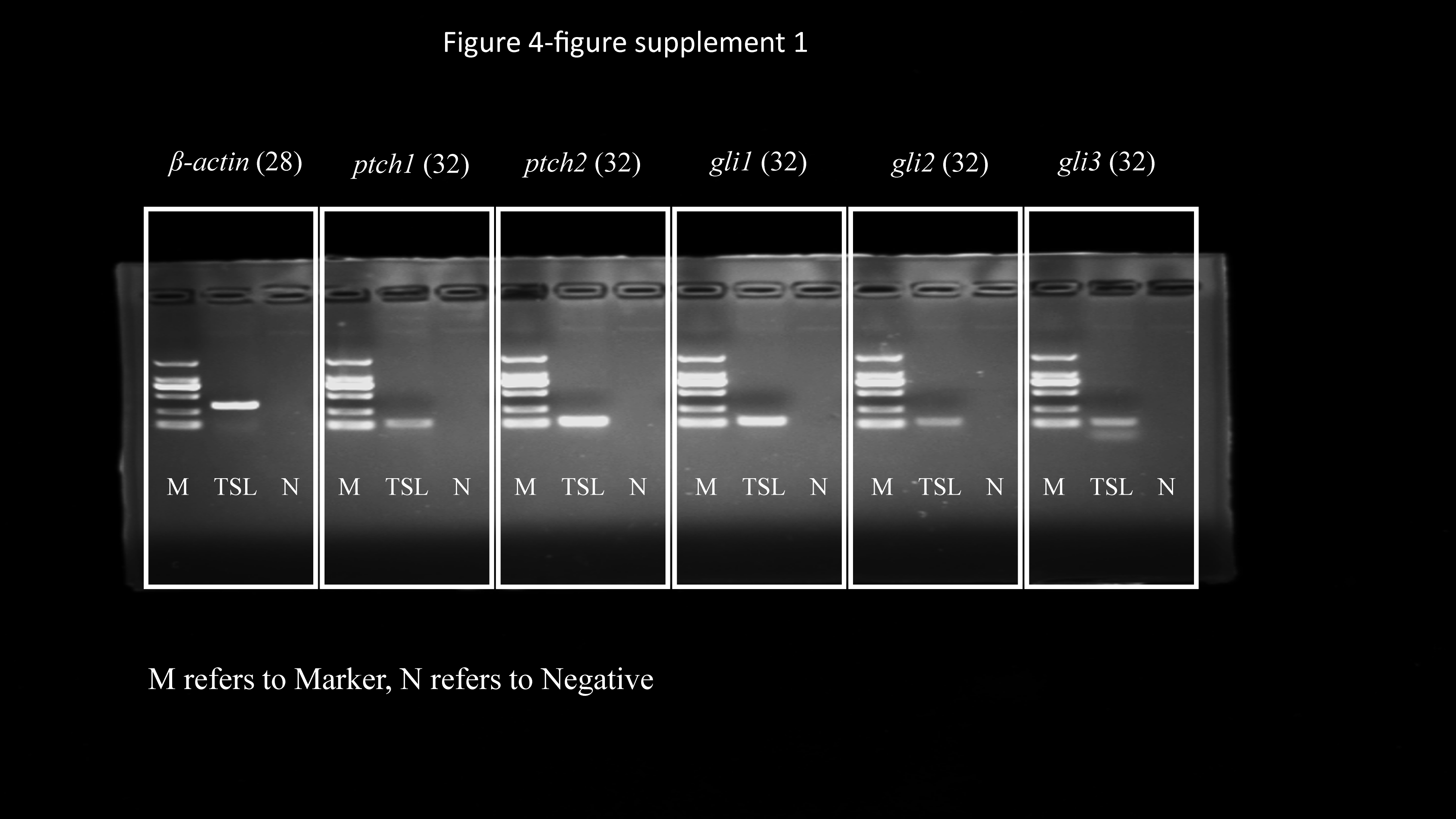

Supplement: Figure 4—figure supplement 1—source data 2. [file elife-109979-fig4-figsupp1-data2.zip › Figure 4-figure supplement 1-source data 2.tif]

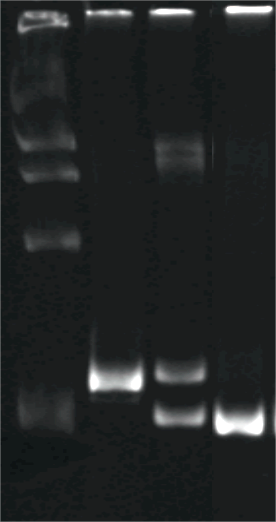

Supplement: Figure 4—figure supplement 3—source data 1. [file elife-109979-fig4-figsupp3-data1.zip › Figure 4-figure supplement 3-source data 1.png]

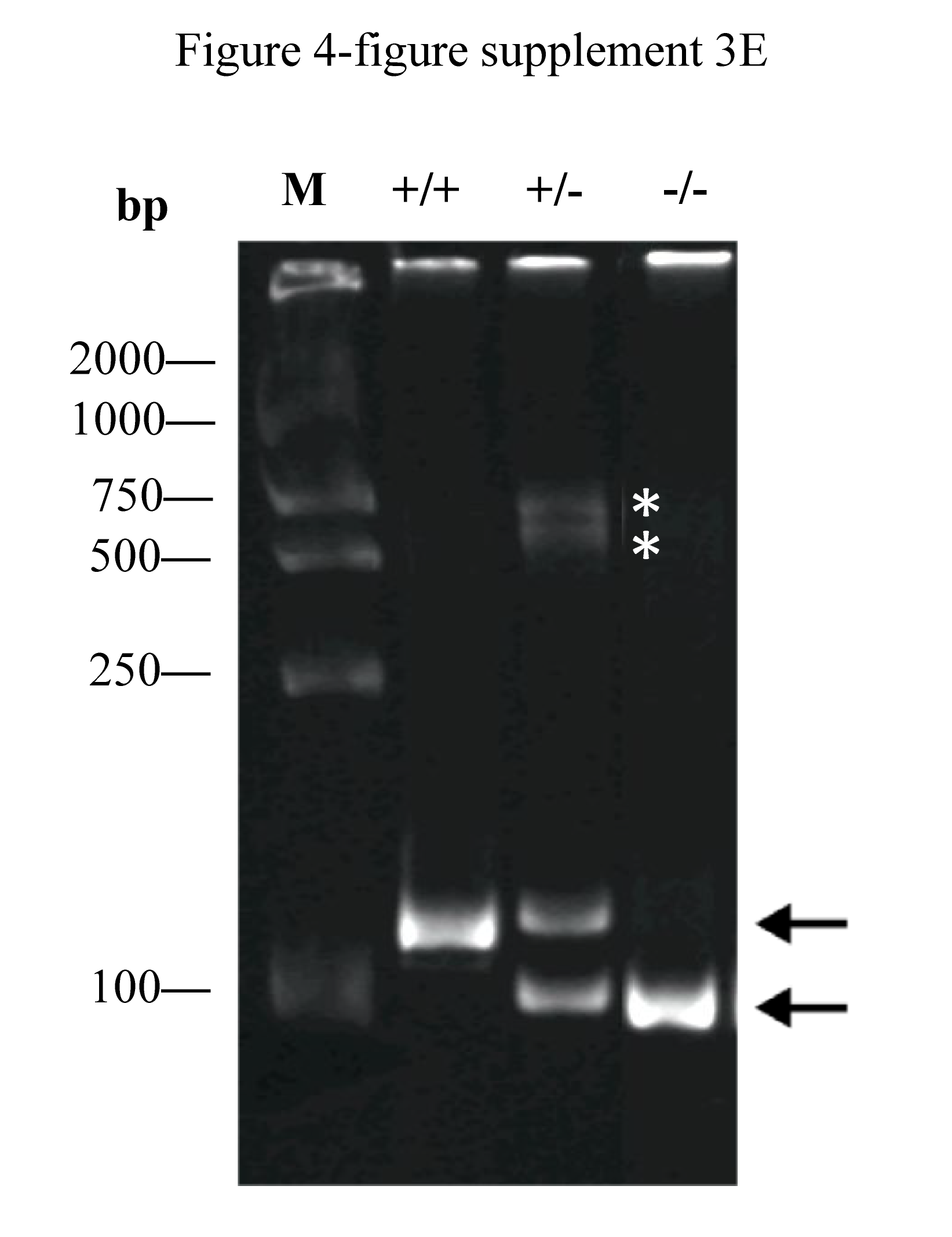

Supplement: Figure 4—figure supplement 3—source data 2. [file elife-109979-fig4-figsupp3-data2.zip › Figure 4-figure supplement 3-source data 2.tif]
