# Supplementary material for: Desert Hedgehog mediates stem Leydig cell differentiation through Ptch2/Gli1/Sf1 signaling axis
Source: eLife. 2026 Jun 29;15:RP109979. doi: 10.7554/eLife.109979 (PMC13313686; doi:10.7554/eLife.109979)
Supplement: Peer review history

# Editor report: eLife Assessment

**DOI:** 10.7554/eLife.109979.3.sa0

**Authors:** Yamini Dalal

**Affiliations:** National Cancer Institute United States

**Keywords:** Convincing, Valuable

This study provides **valuable** contributions to establish canonical Dhh signaling as a primary mediator in the differentiation of Leydig cells and their steroidogenic capacity. Together, the experimental design using their established stem Leydig cell line alongside relevant genetically mutated models, both derived using the relevant Nile tilapia animal system, provided largely **convincing** evidence to support their conclusions. The work will be of broad interest to developmental biologists interested in differentiation of steroidogenic or hormone producing cells.

[Editors' note: this paper was reviewed by Review Commons.]

# Referee report: Reviewer #1 (Public review)

**DOI:** 10.7554/eLife.109979.3.sa1

[Editors' note: this version has been assessed by the Reviewing Editor without further input from the original reviewers. The authors have addressed the comments raised in the previous round of review.]

Summary:

This manuscript by Zhao et. al investigates the canonical hedgehog pathway in testis development of Nile tilapia. They used complementary approaches with genetically modified tilapia and transfected TSL cells (a clonal stem Leydig cell line) previously derived from 3-mo old tilapia. The approach is innovative and provides a means to investigate DHH and each downstream component from the ptch receptors to the gli and sf1 transcription factors. They concluded that Dhh binds Ptch2 to stimulate Gli1 to promote an increase in Sf1 expression leading to the onset of 11-ketotesterone synthesis heralding the differentiation of Leydig cells in the developing male tilapia.'

Strengths of the methods and results:

- The use of Nile tilapia is important as it is an important aquaculture species, it shares the genetic pathway for sex determination of mammalian species, and molecular differentiation pathways are highly conserved

- The approach is rigorous and incorporates a novel TSL, clonal stem Leydig cell model that they developed that is relatively faithful in following endogenous developmental steps and can produce the appropriate steroid.

- Tilapia are relatively amenable to CRISPR/Cas9 targeting and, with their accelerated developmental time frame, provide an excellent model system to interrogate specific signaling pathways.

- The stepwise analysis from dhh-gli-sf1 is thoughtful and well done.

Achieved Aims: The authors set out to test the hypothesis that the canonical Dhh signaling pathway for Leydig cell differentiation and steroidogenic activity is mediated via ptch2 and gli1 regulation of sf1. The results are strong, there are additional steps needed to verify that redundancy/compensation is not contributing to the outcomes.

This work is important in better understanding of nuanced commonalities and differences in developmental pathways across species. Specific to Leydig cell differentiation and steroidogenesis, their work with tilapia supports conservation of the canonical Dhh pathway; however, there appear to be some differences in downstream mediators compared to mouse. Specifically, they conclude that ptch2/gli1 stimulates sf1 and steroidogenesis in tilapia where gli1 is dispensable in mouse. Instead, Gli3 has recently been shown to play an important role to stimulate Sf1 and support the hedgehog pathway.

# Author comment: Author response

**DOI:** 10.7554/eLife.109979.3.sa2

**Authors:** Changle Zhao, Yongxun Chen, Lei Liu, Xiang Liu, Hesheng Xiao, Feilong Wang, Qin Huang, Xiangyan Dai, Wenjing Tao, Deshou Wang, Jing Wei

**Affiliations:** Southwest University Chongqing China; Southwest University Chongqing China; Southwest University Chongqing China; Southwest University Chongqing China; Southwest University Chongqing China; Southwest University Chongqing China; Southwest University Chongqing China; Southwest University Chongqing China; Southwest University Chongqing China; Southwest University Chongqing China; Southwest University Chongqing China

The following is the authors’ response to the original reviews

**Public Reviews:**

**Reviewer #1 (Public review):**

Weaknesses of the methods and results:

- Line 162: need to establish and verify the PKH26-labeled TSL cells were unaffected by the dhh-/- environment. No data to support the claim that they were unaffected.

We thank the reviewer for this important comment. In *dhh*^-/-^ recipient testes, PKH26-labeled TSL cells were observed within the interstitial compartment (Fig. 3C3). Importantly, these PKH26-positive cells could be induced by SAG treatment to differentiate into Cyp11c1-positive steroidogenic cells (Fig. 3E3), indicating that they remained viable in the *dhh*^-/-^ environment.

We have revised the Results section (line 171–173) to “These results suggest that SLC differentiation is inhibited, whereas the survival and engraftment of PKH26-labeled TSL cells were not affected in *dhh*^-/-^ XY tilapia testes.”

- The rescued phenotype caused by the addition of ptch2-/- to the dhh-/- model is a compelling. To further define potential ptch1 contributions, it would be helpful to examine the expression level of ptch1 in the context of the ptch2-/- and ptch2-/-;dhh-/- mutant animals. Any compensatory increase in ptch1 in either case, without obvious phenotype changes, would support the dominant role for ptch2.

We thank the reviewer for this valuable suggestion. We have now performed RT-qPCR analysis of *ptch1* expression in XY testes from WT, *ptch2*^-/-^ and *dhh*^-/-^;*ptch2*^-/-^ fish at 90 dah. As shown in Fig. S8, no significant differences in *ptch1* mRNA levels were detected among these genotypes, indicating that loss of *ptch2* does not induce compensatory upregulation of *ptch1* at the transcriptional level under the conditions examined. We have revised the Discussion section (line 277–290) to “The specificity for Ptch2 in this context might stem from unique co-receptor interactions or expression patterns within the testicular niche. To preliminarily assess potential compensatory regulation, we examined *ptch1* expression in XY testes from WT, *ptch2*^-/-^ and *dhh*^-/-^;*ptch2*^-/-^ fish at 90 dah. No significant differences in *ptch1* mRNA levels were detected among these genotypes (Fig. S8), suggesting that loss of *ptch2* does not trigger compensatory upregulation of *ptch1* at the transcriptional level under the conditions examined. Nonetheless, global *ptch2* mutation affects multiple tissues, whereas our mechanistic focus is on SLC differentiation within the testicular niche. Moreover, the early embryonic lethality of global *ptch1* mutation in tilapia (Liu et al., 2024) precludes direct assessment of its role in postnatal testis development. Therefore, although our findings strongly support a predominant role for Ptch2 in mediating Dhh signaling in SLCs, definitive resolution of receptor specificity will require future Leydig cell-specific conditional knockout models.”

- Activity of individual gli factors need additional reconciliation. The expression profiles for both alternative gli factors should be quantified in each knockout cell line to establish redundancy and/or compensation.

We agree that quantifying the expression of alternative *gli* genes might be informative. In the present study, TSL-*gli1*^-/-^ cells completely lose responsiveness to Dhh stimulation in the 8×GLI luciferase assay, whereas TSL-*gli2*^-/-^ and TSL-*gli3*^-/-^ cells retain normal pathway activation (Fig. 5B), which unambiguously suggest that Gli1 is the principal transcriptional effector in tilapia SLCs under our experimental conditions. Redundancy and/or compensation of alternative *gli* factors need further genetic dissection in the future study.

- Figure 5E: An important control is missing that includes evaluation of HEK293 cells transfected with pcDNA3.1-OnGli1 without the addition of pGL3-sf1.

We don’t think HEK293 cells transfected with pcDNA3.1-OnGli1 without the addition of pGL3-*sf1* is an important control in our study. In the dual-luciferase assays, we think pcDNA3.1 + pGL3 (empty reporter) and pcDNA3.1 + pGL3-*sf1* controls were sufficient.

**Recommendations for the authors:**

**Reviewer #1 (Recommendations for the authors):**

Recommendations for improving the writing and presentation; minor corrections:

- Include Park paper (Endocrinology 2007) somewhere near line 73. Need to acknowledge this paper as it is one of the first to connect Dhh to Sf1.

We have now included the citation of Park *et al*. (Endocrinology 2007) in the Introduction (now line 81).

- Include Kothandapani paper (PLoS Genetics 2020) somewhere near line 86. Need to acknowledge this paper as it is the only to reconcile the data showing no difference in Gli1 or Gli2 knockouts, but loss of Leydig cell function due to Gli3 activity.

We have now included the citation of Kothandapani *et al*. (PLoS Genetics 2020) in the Introduction (now line 97).

- Please include sequences of B1 and B2 in sf1 promoter, how conserved are they to the canonical Gli binding sequence?

We have revised the Results section (line 216–218) to “Functional annotation of its promoter region identified two conserved Gli1-binding motifs, B1 (AACCACCCA) and B2 (GAGCCACCCA)”.

- Figure 1 or results text: please clarify that the dhh-/- model used is the delta13bp mutation.

We have clarified in the Results section (line 133) that the *dhh*^-/-^ model corresponds to the 13-bp (CAGGGATGCGGAC) frameshift deletion.

- Figure 5E legend: please clarify that HEK293 cells are used

We have revised the Figure 5E legend to explicitly state that the dual-luciferase reporter assays were performed in HEK293 cells. Revised legend sentence (line 743-746): HEK293 cells were co-transfected with pRL-TK, pGL3, pcDNA3.1, pGL3-*sf1*, pcDNA3.1-On Gli1, and the indicated cold probe constructs, and luciferase activity was measured 48 hours post-transfection.

- Figure S5E: * indicates the heteroduplex-it seems that there is a heteroduplex highlighted with the asterisk at ~600bp size; based on homozygous and mutant bands, it seems the asterisk should be highlighting the duplex near those sized bands. What are the bands up at ~600bp?

We thank the reviewer for the careful observation. In Figure S5E, the bands observed at approximately ~600 bp represent heteroduplex products formed during the re-annealing of PCR amplicons derived from heterozygous individuals. During denaturation and re-annealing, WT and mutant strands can pair in different configurations, generating distinct heteroduplex conformations that migrate more slowly than homoduplex products in PAGE. As a result, two heteroduplex bands are visible at ~600 bp, reflecting alternative mismatched duplex structures. The homoduplex WT and mutant bands are indicated separately by arrows.

- Figure S7F: dhh-/- data are missing

We thank the reviewer for pointing out this omission. The missing *dhh*^-/-^ dataset has now been added to Figure S7F, and the figure has been updated accordingly.
